# Nonvanishing derived limits without scales

**DOI:** 10.1007/s00153-025-00996-z

**Published:** 2025-11-04

**Authors:** Matteo Casarosa

**Affiliations:** 1 Institut de Mathématiques de Jussieu - Paris Rive Gauche (IMJ-PRG), Université Paris Cité, Bâtiment Sophie Germain, 8 Place Aurélie Nemours, 75013 Paris, France; 2 Dipartimento di Matematica, Università di Bologna, Piazza di Porta S. Donato, 5, 40126 Bologna, Italy

**Keywords:** Derived limits, Strong homology, Cardinal characteristics, Weak diamond, 03E35, 03E17, 03E75, 18E10, 55Nxx

## Abstract

The derived functors $\lim ^n$ of the inverse limit are widely studied for their topological applications, among which are some repercussions on the additivity of strong homology. Set theory has proven useful in dealing with these functors, for instance in the case of the inverse system $\textbf{A}$ of abelian groups indexed over ${}^\omega \omega$. So far, consistency results for nonvanishing derived limits of $\textbf{A}$ have always assumed the existence of a scale (i.e. a linear cofinal subset of $({}^\omega \omega , \le ^*)$, or equivalently that $\mathfrak {b} = \mathfrak {d}$). Here we eliminate that assumption and prove that nonvanishing derived limits, and hence the non-additivity of strong homology, are consistent with any value of $\aleph _1 \le \mathfrak {b} \le \mathfrak {d} < \aleph _\omega$, thus giving a partial answer to a question of Bannister.

## Introduction

This is an article on applications of set theory to homological algebra, and more precisely to the derived functors of the inverse limit. These are usually denoted by $$\lim ^n$$ for $$n > 0 $$ and they may be regarded as measuring failures of the inverse limit functor to be exact.

In recent years, set theory has proven useful to deal with some problems related to derived limits. These problems have repercussions in algebraic topology. For instance, the non-additivity of $\textit{strong homology}$ is implied by the nonvanishing of any of the derived limits $\lim ^n$ of a certain pro-group $\textbf{A}$ indexed on ${}^\omega \omega$ ([12]), while the vanishing of $\lim ^n$ for all *n* for a certain class of pro-groups that generalizes $\textbf{A}$ guarantees the additivity of strong homology on the class of locally compact separable metric spaces ([1, 2]). As it happens, many questions in this area turn out to be independent of the $\textrm{ZFC}$ axioms, and many theorems take the form of consistency results. On the nonvanishing side, the historical progression is as follows:

1. $$\mathfrak {d} = \aleph _1$$ implies that $$\lim ^1 \textbf{A}\ne 0$$ ([8]).
2. $\lim ^1 \textbf{A}^\mathcal {F} \ne 0$ is a $\textrm{ZFC}$-fact, whenever $\mathcal {F}$ is an $\omega _1$-chain in $({}^\omega \omega , \le ^*)$, and $\textbf{A}^\mathcal {F}$ is the restriction of $\textbf{A}$ to the indexing subset $\mathcal {F}$ ([3]).
3. $\lim ^1 \textbf{A}\ne 0$ is consistent with $\textrm{MA}_{\aleph _1}$, (in particular, a witness to its nonvanishing was constructed in a model of $\mathfrak {b} = \mathfrak {d} = \mathfrak {c} = \aleph _2$ [16]). Yet, $\textrm{PFA}$, a strengthening of $\textrm{MA}_{\aleph _1}$, implies $\lim ^1 \textbf{A} = 0$ ([8]).

Then there is a small series of nonvanishing results for higher derived limits, namely, the functors $\lim ^n$ for $n > 1$.

(4) It is consistent that $\lim ^2 \textbf{A}\ne 0$. In particular, it is implied by $\mathfrak {b} = \mathfrak {d} = \aleph _2 + \lozenge (S^1_2)$([4]). For a proof, one starts with the $\textrm{ZFC}$-fact about chains of length $\omega _1$ as a lemma and then deduces the theorem using the guessing principle. Note that the condition $\mathfrak {b} = \mathfrak {d} = \kappa$ is equivalent to the existence of a cofinal $\kappa$-chain in $({}^\omega \omega , \le ^*)$, that is, a $\kappa$-*scale*. This allows us to focus on an $\omega _2$-scale, on which we perform a linear construction. Note also that $\lozenge (S_2^1)$ implies $\mathfrak {c} \le \aleph _2$, and this is the obstruction to using the classical diamonds to get to $\lim ^n \textbf{A}$ for $n>2$.
(5) For all $n >0$ it is consistent that $\lim ^n \textbf{A}\ne 0$. In particular, this is implied by $\mathfrak {b} = \mathfrak {d} = \aleph _n + \bigwedge _{i < n} w\lozenge (S^i_{i+1})$ ([17]). The weak diamonds $w \lozenge (S)$ are some guessing principles that were originally introduced by Devlin and Shelah in [7], and are compatible with a large continuum and longer scales. They are also sufficient to inductively build, starting from the base case of $\lim ^1$ of an $\omega _1$-chain, witnesses to a nontrivial $\lim ^k$ associated with any chain of length $\omega _k$, up to $k=n$

Since all nonvanishing results for this kind of systems so far assume or entail a linear indexing set (or cofinal subset thereof), it is natural to ask what the behavior of the derived limits of $\textbf{A}$ is in case $\mathfrak {b} < \mathfrak {d}$. Indeed, this problem is mentioned among the open questions discussed in a previous version of [1] and in [5]. In this paper, we are going to discuss precisely that question, and more precisely we are going to prove the following.

### Main Theorem

For all $1 \le k \le n < \omega$, it is consistent that $\mathfrak {b}= \aleph _k$, $\mathfrak {d}= \aleph _n$ and $\lim ^n \textbf{A}\ne 0$.

This, in turn, implies (see [12]) the following.

### Corollary 1.1

For all $1 \le k \le n < \omega$, it is consistent that $\mathfrak {b}= \aleph _k$, $\mathfrak {d}= \aleph _n$, and strong homology is not additive on separable locally compact metric spaces.

This is in some ways complementary to the work of Bannister in [1] wherein, by adding $\aleph _\omega$-many Cohen reals over a model of $\textrm{GCH}$, additivity is forced for that same class of spaces.

The structure of the present paper is the following. First, we are going to prove that the existence of an unbounded chain of length $\mathfrak {d}$ in $({}^\omega \omega , \le ^*)$, together with some guessing principles, implies $\lim ^n \textbf{A}\ne 0$. This we prove by induction starting from a base case where we show the presence of coherent families that have some nontriviality concentrated in a particular part of them. This stronger nontriviality condition is then shown to be preserved throughout the induction. This first part generalizes the results of [17] by replacing the assumption on the existence of a cofinal chain of length $\mathfrak {d}$ with that of an unbounded chain of the same length. Finally, we present a forcing that shows the consistency of the assumptions used in the inductive argument with all the relevant values of $\mathfrak {b}$ and $\mathfrak {d}$. This builds upon the results of Hechler, who proved in [9] that for every countably directed well-founded poset there is a forcing that embeds it as a cofinal subset of $({}^\omega \omega , <^*)$. We will use this in particular for the poset $\omega _k \times \omega _n$.

## Preliminaries and notation

Let $\textbf{G} = ( G_{\lambda }, p_{\lambda }^{\mu }, \Lambda )$ be an inverse system of abelian groups, with groups $G_{\lambda }$ indexed over the directed quasi-order $(\Lambda , \le )$ and bonding morphisms $p^{\mu }_{\lambda }: G_{\mu } \rightarrow G_{\lambda }$, with $\lambda \le \mu \in \Lambda$, between them. Let moreover $\Lambda ^{(n)}$ be the set of $\le$-ordered $(n+1)$-tuples in $\Lambda$. If $\bar{\lambda } = (\lambda _0,..., \lambda _n ) \in \Lambda ^{(n)} \,$we write $\bar{\lambda ^i} = (\lambda _0,..., \lambda _{i-1}, \widehat{\lambda _i}, \lambda _{i+1},..., \lambda _n)$, meaning that we have removed the element $\lambda _i$.

The derived limit $\lim ^n$, for $n > 0$, is the *n*-th derived functor of the inverse limit $\lim$, also denoted by $\lim ^0$. We refer the reader interested in the definition of derived functors through resolutions or the total derived functor to [18] and [15] respectively. Throughout the proof of the main theorem, we will use the following characterization due to Roos ([14]).

### Definition 2.1

(Roos complex) We define the cohomological Roos complex as

$$\begin{aligned} K^{\bullet } (\textbf{G}) \, = \, ( \, 0 \rightarrow K^0 (\textbf{G}) \xrightarrow []{\delta ^1} K^1 (\textbf{G}) \xrightarrow []{\delta ^2} K^2 (\textbf{G}) \xrightarrow []{\delta ^3} ... \, ), \end{aligned}$$

where

$$\begin{aligned} K^n (\textbf{G}) = \prod _{\bar{\lambda } \in \Lambda ^{(n)} } G_{\lambda _0 } = \prod _{\lambda _0 \le ... \le \lambda _n } G_{\lambda _0}. \end{aligned}$$

In other words, $$K^n $$ consists of all functions $$\bar{x}: \Lambda ^{(n)} \rightarrow \bigcup _{\lambda } G_{\lambda } $$ (we think of the $$G_{\lambda }$$ as disjoint) such that $$\bar{x} (\bar{\lambda }) \in G_{\lambda _0} $$. We will also write $$x_{\bar{\lambda }}$$ for $$\bar{x} (\bar{\lambda }) $$ and $$\textbf{G}^{(n)}$$ for $$K^n(\textbf{G})$$ and $$\textbf{G}^{(n)} \restriction C$$ for $$K^n(\textbf{G}\restriction C)$$. The coboundary maps are

$$\begin{aligned} \delta ^n (\bar{x})_{\bar{\lambda }} = p_{\lambda _0}^{\lambda _1} (x_{\bar{\lambda ^0}} ) + \sum _{1 \le i \le n} (-1)^i x_{\bar{\lambda ^i}}. \end{aligned}$$

Note that we stipulate that $K^{-1} (\textbf{G}) = 0$ and $\delta ^0 = 0$.

We also define a couple of terms related to this construction.

### Definition 2.2

(Coherent and trivial elements) We call an element *n*-*coherent* if it is in the kernel of $\delta ^{n+1}$, and we call it *n*-*trivial* if it is in the image of $\delta ^n$. If $\delta ^n (\bar{y}) = \bar{x}$ we say that $\bar{y}$ *trivializes* $\bar{x}$.

The derived limits can be computed as the cohomology of the Roos complex.

### Theorem 2.3

Let $\textbf{G}$ be an inverse system of groups, $K^\bullet$ be the associated Roos complex, and $n \ge 0$. Then

$$ \lim ^n \textbf{G}\cong H^n (K^\bullet ) $$.

This is part of a theorem for which we refer the reader to [11, §11.5].

We now record two basic results we will need in the next section.

### Definition 2.4

Let

$$\begin{aligned} {\textbf {G}} = \lbrace G_{\lambda } ; p^{\mu }_{\lambda } : \lambda \le \mu \in \Lambda \rbrace . \end{aligned}$$

be an inverse system of abelian groups. We say that the system is surjective if each $p^{\mu }_{ \lambda }$ is surjective.

### Theorem 2.5

(Surjective Goblot’s Theorem [17]) Let $n \ge 0$. If $\textbf{G}$ is a surjective inverse system of abelian groups with $\text {cof} ( \textbf{G}) = \aleph _n$, then $\lim ^k \textbf{G}= 0$, for all $k \ge n+1$.

If *M* is a directed subset of the indexing set $\Lambda$ we will write $\textbf{G}\restriction M$ for the system $\lbrace G_{\lambda }; p^{\mu }_{\lambda }: \lambda \le \mu \in M \rbrace$.

### Theorem 2.6

(Theorem B of ([13])) Let $\textbf{G}$ be an inverse system indexed over $\Lambda$, let *M* be another directed set and let $\phi : M \rightarrow \Lambda$ be an order preserving cofinal map. Let moreover $\phi ^*(\textbf{G})$ be the system indexed on *M* defined by pre-composing the functor $\phi$ between the two orders thought of as categories to the functor $\textbf{G}$. Then, for all $n \ge 0$,

$$ \lim ^n \textbf{G}\cong \lim ^n \phi ^* (\textbf{G}). $$

In particular, the case where $\phi$ is an inclusion gives us that for a cofinal subset $C \subseteq \Lambda$, we have $\lim ^n \textbf{G}\cong \lim ^n \textbf{G}\restriction C$.

To more easily apply the last two theorems we will introduce the setting of quotient systems described in the following section and will apply it to the systems in which we are interested.

## The inverse system $\textbf{A}$ and its relatives

The main object of our interest is the inverse system $\textbf{A}$. It is indexed over the set ${}^\omega \omega$ of all functions $f: \omega \rightarrow \omega$ and such that the object $A_f$ corresponding to *f* is defined as

$$\begin{aligned} A_f = \bigoplus _{i \in \omega } \mathbb {Z}^{f(i)}. \end{aligned}$$

The order relation on this set is everywhere domination (i.e., $f \le g$ if and only if $f(i) \le g(i)$ for all $i \in \omega$). The bonding morphisms are the obvious projections. We write $I_f = \lbrace (i, j) \mid j \le f(i) \rbrace$ for the region on which the elements of the groups seen as functions to $\mathbb {Z}$ are defined. Analogously, we define the system $\textbf{B}$ by replacing infinite sums with infinite products:

$$\begin{aligned} B_f = \prod _{i \in \omega } \mathbb {Z}^{f(i)}. \end{aligned}$$

This yields a short exact sequence of inverse systems

$$\begin{aligned} 0 \rightarrow \textbf{A}\rightarrow \textbf{B}\rightarrow \textbf{B}/ \textbf{A}\rightarrow 0, \end{aligned}$$

which induces a long exact sequence

$$ 0 \rightarrow ... \rightarrow \lim ^n \textbf{A}\rightarrow \lim ^n \textbf{B}\rightarrow \lim ^n \textbf{B}/ \textbf{A}\rightarrow \lim ^{n+1} \textbf{A}\rightarrow ... \,\,\,\,\,\, . $$

Now, [12, Lemma 4] says that $\lim ^n \textbf{B}= 0$ for $n \ge 1$, so that $\lim ^{n+1} \textbf{A}\cong \lim ^n \textbf{B}/ \textbf{A}$ and $\lim ^1 \textbf{A}\cong \frac{\lim ^0 \textbf{B}/ \textbf{A}}{\lim ^0 \textbf{B}}$. The proof straightforwardly generalizes to restrictions to directed subsets and groups different than $\mathbb {Z}$. In other words, for any directed $\mathcal {F} \subseteq {}^\omega \omega$, and any nontrivial abelian group *G*, we can define $\textbf{A}^{\mathcal {F}}_G$ and $\textbf{B}^{\mathcal {F}}_G$ whose objects are $A_f = \bigoplus _{i \in \omega } G^{f(i)}$ and $B_f = \prod _{i \in \omega } G^{f(i)}$ respectively, for each $f \in \mathcal {F}$. Then the reasoning above still yields isomorphisms $\lim ^1 \textbf{A}^\mathcal {F}_G \cong \frac{\lim ^0 \textbf{B}^\mathcal {F}_G / \textbf{A}^\mathcal {F}_G}{\lim ^0 \textbf{B}^\mathcal {F}_G}$ and $\lim ^{n+1} \textbf{A}^\mathcal {F}_G \cong \lim ^n \textbf{B}^\mathcal {F}_G / \textbf{A}^\mathcal {F}_G$.

For the case $n > 1$, we further observe that the inclusion map $\iota : (\mathcal {F}, \le ) \hookrightarrow (\mathcal {F}, \le ^*)$ is order-preserving and cofinal, hence by Theorem 2.6 we can further substitute for $\lim ^n \textbf{B}^\mathcal {F}_G / \textbf{A}^\mathcal {F}_G$ the limit $\lim ^n (\textbf{B}^\mathcal {F}_G / \textbf{A}^\mathcal {F}_G)_{\le ^*}$ of a system with the same quotient groups as objects and with bonding morphisms analogous to the previous ones for each instance of $\le ^*$. One can justify the same conclusions by the approach of [17]. This last system is surjective and has the property of being indexed on the very order whose cofinality is by definition $\mathfrak {d}$. This will be relevant for the use we are going to make of Theorem 2.5.

Let $=^*$ stand for equality except possibly on finitely many points. The isomorphism for $\lim ^1$ yields the following characterization, where one can indifferently use $\omega \times \omega$ or $\bigcup _{f \in \mathcal {F}} I_f$ as the domain of $\psi$.

### Proposition 3.1

Let *G* be a nontrivial group, and let $\mathcal {F} \subseteq {}^\omega \omega$ be directed. Then $\lim ^1 \textbf{A}^\mathcal {F}_G \ne 0$ if and only if there exists a family $\langle \phi _f: I_f \rightarrow G \mid f \in \mathcal {F}\rangle$ of functions such that:

1. For every two $f,g \in \mathcal {F}$, $\phi _f \restriction I_f \cap I_g =^*\phi _g \restriction I_f \cap I_g$.
2. There exists no function $\psi : \omega \times \omega \rightarrow G$ such that $\psi \restriction I_f =^*\phi _f$ for all $f \in \mathcal {F}$.

We call a family that satisfies the first condition *coherent*. If it satisfies the second condition we say moreover that it is *nontrivial*.

### Remark 3.2

Note that $\lim ^1 \textbf{A}^\mathcal {F}_G \ne 0$ implies $\lim ^1 \textbf{A}^\mathcal {F}_{G'} \ne 0$ whenever $\vert G \vert \le \vert G' \vert$, so that the vanishing of $\lim ^1 \textbf{A}^\mathcal {F}_G$ is equivalent for groups of the same cardinality, while the group structure is relevant for higher limits. This is because the equation $a-b= 0$ reduces to the logical notion of equality $a=b$, so that a witness for $\lim ^1 \textbf{A}^\mathcal {F}_G \ne 0$ can be seen modulo injection as a witness for $\lim ^1 \textbf{A}^\mathcal {F}_{G'} \ne 0$, for any $G'$ of greater or equal size, in contrast, there is an inescapable algebraic aspect to longer alternating sums.

The characterization for higher derived limits can be expressed in terms of *n*-coherent and *n*-nontrivial families for the quotient system.

### Proposition 3.3

For $n\ge 1$, $\lim ^{n+1} \textbf{A}^\mathcal {F}_G \ne 0$ if and only if there exists $\bar{x} \in ((\textbf{B}^\mathcal {F}_G / \textbf{A}^\mathcal {F}_G)_{\le ^*})^{(n)}$ such that $\delta ^{n+1} ( \bar{x} ) = 0$ and such that for no $\bar{y} \in ((\textbf{B}^\mathcal {F}_G / \textbf{A}^\mathcal {F}_G)_{\le ^*})^{(n-1)}$ we have $\delta ^n( \bar{y} ) = \bar{x}$.

We note however that the established usage is to refer to these witnesses (or, more precisely, their representatives in $\textbf{B}^{\mathcal {F}}_G$ as nontrivial $(n+1)$-coherent families, since they witness a nontrivial $\lim ^{n+1} \textbf{A}^{\mathcal {F}}_G$.

## Unbounded nontriviality

Before we start the proof by induction of the main result of this section, we need a lemma.

### Definition 4.1

Let $P \subseteq {}^\omega \omega$. We say that $C \subseteq P$ is a $\kappa$-*chain* if there is a bijection $\sigma : \kappa \rightarrow C$ such that $\alpha \le \beta$ implies $\sigma (\alpha ) \le ^*\sigma (\beta )$.

### Remark 4.2

If $\kappa$ is regular and *C* is unbounded in *P*, then we can find $C' \subseteq C$ cofinal in *C*, ordered by the strict order $\prec$ defined as $f \prec g \leftrightarrow (f \le ^*g \wedge \lnot (f \ge ^*g))$.

### Lemma 4.3

Let $n \ge 0$ and $P \subseteq {}^\omega \omega$ be such that $\text {cof} (P, \le ^*) = \aleph _{n+1}$ and there exists an $\omega _{n+1}$-chain *C* that is unbounded in *P*. Let moreover *P* be $\vee$-closed (here $\vee : ({}^\omega \omega )^2 \rightarrow {}^\omega \omega$ is defined as $(f \vee g)(i) = \max \lbrace f(i), g(i) \rbrace$) and $\le ^*$-downward closed.

Then we can find a continuous increasing union

$$\begin{aligned} P = \bigcup _{\alpha < \omega _{n+1}} P_\alpha , \end{aligned}$$

and a $C' \subseteq C$ cofinal in *C* such that for each $\alpha < \omega _{n+1}$, $P_\alpha$ is $\vee$-closed, $\le ^*$-downward closed, with $\text {cof} (P_\alpha ) \le \aleph _n$, $\textrm{otp}(P_\alpha \cap C') = \alpha$ and $P_\alpha \cap C'$ unbounded in $P_\alpha$ for every limit $\alpha$.

### Proof

Let $c: \omega _{n+1} \rightarrow P$ be an enumeration of a cofinal subset of *P*, and assume without loss of generality, thanks to Remark 4.2, that *C* is a chain of order-type $\omega _{n+1}$ for the strict order relation defined therein.

We construct by mutual transfinite recursion the increasing union and a function $b: \omega _{n+1} \rightarrow C$ such that $b(\alpha )$ is the least element of $C \setminus P_\alpha$, where $P_\alpha$ is the downward closure of the $\vee$-closure of $c[\alpha ] \cup b[\alpha ]$ (note that this stays $\vee$-closed). This is increasing and continuous by construction. The $\vee$-closure of $c[\alpha ] \cup b[\alpha ]$ is cofinal in $P_\alpha$ and of cardinality $\le \aleph _n$. For each element in it, we can find one in the chain that is not dominated by it; their supremum (which exists because the chain has cofinality $> \aleph _n$) will not be in the downward-closure $P_\alpha$. This shows that *b* is well-defined, strictly increasing, and hence cofinal in *C*. Finally, we put $C' = b[\omega _{n+1}]$. The conditions on the order-type and unboundedness are immediate by construction. $\square$

### Remark 4.4

Note that, for every limit $\lambda$, $\text {cof} (P_\lambda ) \ge \text {cof} (\lambda )$. This follows from the fact that for every limit $\lambda < \omega _{n+1}$, $C' \cap P_\lambda$ is unbounded in $P_\lambda$. Otherwise, for each element in a cofinal set of lesser cardinality we pick one of the chain that is not dominated by it, and taking the supremum we get a contradiction, as in the previous proof.

The proof in [17] served as a basis for the following argument. The central idea allowing the present advances is the construction of some nontriviality for the base case having the additional property of being “localized", meaning that the restriction of a larger coherent family to a certain chain is nontrivial, and the observation that this additional property is preserved throughout the induction.

### Base case

Now we prove the base case of our induction for every nontrivial abelian group *G* as the codomain of our functions, even though some of this generality will be lost in the inductive step. For this we use a cardinal arithmetic assumption. We do not know whether this assumption is necessary to prove the result in all its generality.

#### Lemma 4.5

Assume that $2^{\aleph _0} < 2^{\aleph _1}$. Let $P \subseteq ({}^\omega \omega , \le ^*)$ be $\vee$-closed and such that $\text {cof} (P) = \aleph _1$ and there exists an $\omega _1$-chain *C* which is unbounded in *P*, and let $G \ne 0$ be an abelian group. Then there is a coherent 1-family $\Phi = \langle \phi _f: I_f \rightarrow G \mid f \in P \rangle$ such that $\Phi \restriction C$ is (coherent) nontrivial.

#### Proof

By the reasoning that shows Remark 3.2, we can restrict to the case $G = \mathbb {Z}/2\mathbb {Z}$.

Apply Lemma 4.3 to get a continuous increasing union $\bigcup _{\alpha < \omega _1} P_\alpha = P$ and $C'$ satisfying the lemma. Recall in particular that the $\alpha$-th element of $C'$ (henceforth $g_\alpha$) is not in the downward-closed set $P_\alpha$, and $\text {cof} (P_\alpha ) \le \omega$. So let $\lbrace \hat{f}_{\alpha ,n} \mid n < \omega \rbrace$ be a cofinal subset of $P_\alpha$. Since $P_\alpha$ is $\vee$-directed we can let $f_{\alpha ,n} = \bigvee _{i \le n} \hat{f}_{\alpha , i}$ so that $\langle f_{\alpha , n} \mid n < \omega \rangle$ is cofinal for $\le ^*$ and a chain with respect to $\le$.

Since $g_\alpha \not \le ^*f_{\alpha ,n}$, the set $I_{g_\alpha } \setminus I_{f_{\alpha ,n}}$ is infinite (in particular non-empty) for all *n*. Pick $x_{\alpha , n} \in I_{g_\alpha } \setminus I_{f_{\alpha , n}}$. Then $X_\alpha = \lbrace x_{\alpha , n} \rbrace _{n < \omega } \subseteq I_{g_\alpha }$ is infinite and almost disjoint from all of the $I_{f_{\alpha , n}}$ as $X_\alpha \cap I_{f_{\alpha , n}} \subseteq \lbrace x_{\alpha , 0},..., x_{\alpha , n-1} \rbrace$.

We now define a tree of coherent families ordered by end-extension, such that among the unions of the branches one yields a nontrivial family by counting arguments.

For all $$s \in 2^{< \omega _1} $$ we iteratively construct a coherent family $$\Phi ^s$$ on $$P_\alpha $$, where $$\text {dom}(s) =\alpha $$ as follows. If $$\alpha \in \lim (\omega _1)$$, then $$\Phi ^s = \bigcup _{\alpha < \text {dom}(s)} \Phi ^{s \restriction \alpha }$$. Suppose now that $$\alpha = \beta +1$$, we have already defined $$\Phi ^s$$ with $$\text {dom}(s) = \beta $$ and we want to define $$\Phi ^{s, 0}$$ and $$\Phi ^{s,1}$$. So we find a trivialization $$\psi ^s$$ of $$\Phi ^s$$ that exists since $$P_\beta $$ is of countable cofinality, so we can consider its cofinal chain $$\langle f_{\beta , n} \mid n < \omega \rangle $$ defined above; it is then easy to see that we can inductively find finite sets $$F_n $$ such that for all $$m \le n $$ we have that $$\phi ^s_{f_{\beta , m}} \restriction (I_{f_{\beta , m}} \setminus F_m )$$ and $$ \phi ^s_{f_{\beta , n}} \restriction (I_{f_{\beta , n}} \setminus F_n )$$ coincide on their common domain. We can then define

$$ \psi ^s (x) = {\left\{ \begin{array}{ll} \phi ^s_{f_{\beta , n}} \,\,\,\, \text {if} \, x \in (I_{f_{\beta ,n}} \setminus F_n ) \,\, \text {for some} \, n \\ 0 \,\,\,\, \text {otherwise}. \end{array}\right. } $$

Then we let $\psi ^{s,0} = \psi ^s$ and

$$ {\left\{ \begin{array}{ll} \psi ^{s,1} (x) = \psi ^s (x) +1 \,\,\,\, \text {if} \, x \in X_\beta \\ \psi ^s (x) \,\,\,\, \text {otherwise}. \end{array}\right. } $$

Finally, we let $\Phi ^{s,0} = \delta ^1(\psi ^{s,0})$ and $\Phi ^{s,1} = \delta ^1 (\psi ^{s,1})$.

Now for all $h: \omega _1 \rightarrow 2$ we put $\Phi ^h = \bigcup _{\alpha < \omega _1} \Phi ^{h \restriction \alpha }$. Note that if $h \ne h'$, then $\phi ^h_{g_\gamma } \ne ^*\phi ^{h'}_{g_\gamma }$ where $\gamma$ is such that $h(\gamma ) \ne h'(\gamma )$. But then the same trivialization cannot trivialize both $\Phi ^h \restriction C'$ and $\Phi ^{h'} \restriction C'$. Since there are only $2^{\aleph _0}$ many putative trivializations and we are assuming that $2^{\aleph _0} < 2^{\aleph _1}$, there must be $\bar{h}: \omega _1 \rightarrow 2$ such that $\Phi ^{\bar{h}} \restriction C'$ is nontrivial. Hence, by Theorem 2.6, $\Phi ^{\bar{h}} \restriction C$ is also coherent nontrivial. $\square$

#### Remark 4.6

In case *G* is infinite, the cardinal arithmetic assumption $2^{\aleph _0} < 2^{\aleph _1}$ is not needed, as one can simply apply the reasoning of [17, Proposition 3.2] with the sets $X_\alpha$ above instead of the sets $b_\xi$ of the cited paper, after fixing a bijection between $\omega \times \omega$ and $\omega$, and an injection of the latter into *G*.

### Induction step

#### Definition 4.7

We say that *R*(*n*, *G*) holds for some group *G* if for every $\vee$-closed $P_n \subseteq ({}^\omega \omega , \le ^*)$ with $\text {cof} (P_n) = \aleph _n$ that contains an $\omega _n$-chain $C_n$ that is unbounded in it, there exists an *n*-coherent family on $P_n$ for *G* such that its restriction to $C_n$ is nontrivial.

#### Remark 4.8

By Proposition 3.1 and Proposition 3.3, *R*(*n*, *G*) implies in particular $\lim ^n \textbf{A}^{P_n}_G \ne 0$ for every $P_n$ as in the definition above.

Whenever *S* is a stationary set, $w \lozenge (S)$ denotes a guessing principle called *weak diamond* of *S*, introduced in [7]. Recall that $S^n_{n+1} = \lbrace \alpha < \omega _{n+1} \mid \text {cof} (\alpha ) = \omega _n \rbrace$.

#### Lemma 4.9

Suppose $$n \ge 1$$, $$\vert G \vert \le 2^{\aleph _1}$$, and both *R*(*n*, *G*) and $$w \lozenge (S^n_{n+1})$$ hold. Then $$R(n+1, G) $$ holds.

#### Proof

Let *P* be as in the statement of $R(n+1, G)$. Using Lemma 4.3 we can write $P = \bigcup _{\alpha < \omega _{n+1}} P_\alpha$ with further assumptions on the cofinality of the latter sets and their relation to the unbounded chain. Throughout the proof, we are going to use the quotient system discussed in section 3, so for any $f \in P$ we define $Q_f = \prod _{i \in \omega } G^{f(i)} / \bigoplus _{i \in \omega } G^{f(i)}$ and let $p^{f'}_f: Q_{f'} \rightarrow Q_f$ for the natural projection whenever $f \le ^*f'$. Finally, let

$$\begin{aligned} \textbf{Q}= \lbrace Q_f ; p^{f'}_f ; P \rbrace . \end{aligned}$$

Let $T = \lbrace s \in 2^{< \omega _{n+1}} \mid \text {supp}(s) \subseteq S^n_{n+1} \rbrace$ with the ordering being end-extension. For $s \in T$ we let $r(s) = P_{\text {dom}(s)}$.

We now define, for all $s \in T$, a sequence $\bar{x} ^s$ such that:

1. $\bar{x} ^s \in \textbf{Q}\restriction r(s)^{(n)}$ and is coherent.
2. If $t \ge s$ then $\bar{x} ^t$ extends $\bar{x} ^s$.
3. If $\text {cof} ( \text {dom}(s)) = \aleph _n$ and $\bar{u} \in \textbf{Q}\restriction ( C \cap r(s))^{(n-1)}$ trivializes $\bar{x} ^s \restriction ( C \cap r(s) )^{(n)}$ then there exists $\epsilon \in \lbrace 0, 1 \rbrace$ such that there is no extension of $\bar{u}$ to a trivialization of $\bar{x} ^{s, \epsilon } \restriction (C \cap P_{\text {dom}(s) +1} )^{(n)}$.

If $\text {dom}(s)$ is limit, and $\bar{x} ^t$ has already been constructed for all *t* with $\text {dom}(t) < \text {dom}(s)$, we let $\bar{x} ^s = \bigcup _{\alpha < \text {dom}(s) } \bar{x} ^{s \restriction \alpha }$.

Now for the successor step, since $\text {cof} (r(s)) \le \aleph _n$ then $\bar{x} ^s \in \textbf{Q}\restriction r(s)^{(n)}$ is trivial by Goblot (Theorem 2.5). Then for $\epsilon \in \lbrace 0,1 \rbrace$, let $s, \epsilon = s^\frown \lbrace \epsilon \rbrace$ and $\bar{w} ^s \restriction r(s)^{(n-1)}$ be a trivialization of $\bar{x} ^s$ as in the induction, and $\bar{v} ^s \in \textbf{Q}\restriction (r(s, 0) )^{(n-1)}$ be

$$ \bar{v} ^s_{\bar{\gamma } } = {\left\{ \begin{array}{ll} \bar{w} ^s_{\bar{\gamma }} \,\,\,\,\, \text {if} \,\,\, \bar{\gamma } \in r(s)^{(n-1)} \\ 0 \,\,\,\,\,\,\, \text {if} \,\,\, \bar{\gamma } \in (r(s, 0) )^{(n-1)} \setminus r(s)^{(n-1)}. \end{array}\right. } $$

Finally, if $\text {cof} (\text {dom}(s)) < \aleph _n$ we let $\bar{x} ^{s, 0} = \delta ^n ( \bar{v} ^s )$.

Otherwise, $\aleph _n = \text {cof} (\text {dom}(s) ) \le \text {cof} (r(s)) \le \aleph _n$. In that case, we pick a family $\bar{z} ^s \in \textbf{Q}\restriction r(s)^{(n-1)}$ that witnesses the induction hypothesis, that is, it is coherent on $P_{\text {dom}(s)}$ and nontrivial even when restricted to the $\omega _n$-chain unbounded in it that corresponds to a witness of the cofinality of $\text {dom}(s)$ (or equivalently to its $\le ^*$-closure) and let $\bar{y} ^s \in \textbf{Q}\restriction (r(s, 0) )^{(n-1)}$

$$ \bar{y} ^s_{\bar{\gamma } } = {\left\{ \begin{array}{ll} \bar{z} ^s_{\bar{\gamma }} \,\,\,\,\, \text {if} \,\,\, \bar{\gamma } \in r(s)^{(n-1)} \\ 0 \,\,\,\,\,\,\, \text {if} \,\,\, \bar{\gamma } \in (r(s, 0) )^{(n-1)} \setminus r(s)^{(n-1)}. \end{array}\right. } $$

Finally, we let: $\bar{x} ^{s, 0} = \delta ^n ( \bar{v} ^s )$ and $\bar{x} ^{s, 1} = \delta ^n ( \bar{v} ^s + \bar{y} ^s )$.

Let us now check that conditions (1) to (3) are satisfied. An increasing union of coherent sequences is coherent, and it extends the sequences of which it is a union, so conditions 1 and 2 are preserved at limit steps. They are also preserved at successor steps because trivial sequences are coherent and

$$\begin{aligned} \delta ^n ( \bar{v} ^s + \bar{y} ^s) \restriction r(s)^{(n)} = \delta ^n ( \bar{v} ^s) \restriction r(s)^{(n)} + \delta ^n ( \bar{y} ^s) \restriction r(s)^{(n)} = \delta ^n ( \bar{v} ^s) \restriction r(s)^{(n)} = \end{aligned}$$

$$\begin{aligned} = \delta ^n ( \bar{v} ^s \restriction r(s)^{(n)} ) = \delta ^n ( \bar{w} ^s) = \bar{x} ^s. \end{aligned}$$

The only thing left is to prove condition 3. Suppose by contradiction that $\bar{u} ^0 \restriction (C \cap r(s) )^{(n-1)} = \bar{u} = \bar{u} ^1 \restriction ( C \cap r(s) )^{(n-1)}$, $\delta ^n ( \bar{u} ^0) = x^{s, 0} \restriction ( C \cap r(s,0)) )^{(n)}$ and $\delta ^n ( \bar{u} ^1) = x^{s, 1} \restriction (C \cap r(s,0) ) )^{(n)}$.

Let $f = \max \lbrace C \cap r(s,0) \rbrace$ and $\bar{u} \in \textbf{Q}\restriction P_{\text {dom}(s)+1}^{(n-1)}$. We define $d_f( \bar{u} ) \in \textbf{Q}\restriction (C \cap r(s))^{(n-2)}$ by $( d_f ( \bar{u} ))_{\bar{h}} = \bar{u} _{\bar{h}, f }$, Then, if $\bar{g}$ ranges over $(C \cap r(s))^{(n-1)}$, we have (omitting restrictions for readability):

$$\begin{aligned} \delta ^{n-1} ( d_f ( \bar{u} ^0) )_{\bar{g}}= &   \sum _{0\le i \le n-1} (-1)^i (d_f \bar{u} ^0)_{\bar{g}^i} = \sum _{0\le i \le n-1} \bar{u} ^0_{\bar{g}^i, f} \\= &   (-1)^{n-1} \bar{u} ^0_{\bar{g}} + \bar{x} ^{s, 0}_{\bar{g}, f} = (-1)^{n-1} \bar{u} ^0_{\bar{g}} + (-1)^n \bar{w} _{\bar{g}}. \end{aligned}$$

By the same reasoning

$$\begin{aligned} \delta ^{n-1} ( d_f ( \bar{u} ^1) )_{\bar{g}} = (-1)^{n-1} \bar{u} ^0_{\bar{g}} + (-1)^n \cdot ( \bar{w} _{\bar{g}} + \bar{z} ^s_{\bar{g}} ), \end{aligned}$$

so that, since $\bar{u} ^0$ and $\bar{u} ^1$ agree on $(C \cap r(s))^{(n-1)}$

$$\begin{aligned} \delta ^{n-1} ( d_f ( \bar{u} ^1 - \bar{u} ^0 ) ) = (-1)^n \bar{z} ^s \restriction (C \cap r(s))^{(n-1)}, \end{aligned}$$

contradicting the fact that the restriction of $\bar{z} ^s$ to the unbounded chain is nontrivial.

It should be noticed that the case $n=1$ is computationally identical but conceptually different insofar as we need to stipulate that a family indexed by 0-tuples is a singleton and that a function defined on $I_f$ can be regarded as a trivialization of a 1-family below *f* as it can be extended to the entire grid $\omega \times \omega$.

This concludes the construction of the $\bar{x} ^s$ for $s \in T$. For $g \in 2^{\omega _{n+1}}$ with $\text {supp}(g) \subseteq S^n_{n+1}$ let

$$\begin{aligned} \bar{x} ^g = \bigcup _{\lambda < \omega _{n+1} } \bar{x} ^{g \restriction \lambda }. \end{aligned}$$

Note that $\bar{x} ^g \in \textbf{Q}^{(n)}$ is coherent (increasing union of coherent sequences).

Now we need a club $D \subseteq \omega _{n+1}$ such that for all $\lambda \in D$ and $s \in T$ with $\text {dom}(s) = \lambda$, the latter codes a sequence $\bar{u} ^s \in \textbf{Q}\restriction (C \cap r(s))^{(n-1)}$ and moreover:

- If $\lambda \in D$ then every $\bar{u} ^s \in \textbf{Q}\restriction (C \cap r(s))^{(n-1)}$ is coded by some $s: \lambda \rightarrow 2$.
- If $s \le t$ then $\bar{u} ^t$ extends $\bar{u} ^s$.

For this, we will use

$$\begin{aligned} D = \lbrace \lambda < \omega _{n+1} \mid \omega _n \cdot \lambda = \lambda \rbrace , \end{aligned}$$

since for any two elements $\lambda , \lambda ' \in D$ there is a subset of order-type at least $\omega _n$ between them in $\omega _{n+1}$. Now let $\lambda _+$ be the successor of $\lambda$ in the increasing enumeration of the elements of *D*. Then there are enough end-extensions of $s: \lambda \rightarrow 2$ to an element in $2^{\lambda _+}$ to code all the different extensions of $\bar{u} ^s \in \textbf{Q}\restriction (C \cap P_{\lambda })^{(n-1)}$ to a sequence $\bar{u} ^t \in \textbf{Q}\restriction (C \cap P_{\lambda _+})^{(n-1)}$. Indeed, these are at most $(\vert G \vert ^{\aleph _0})^{\aleph _n} \le 2^{\aleph _n}$. The base case of the transfinite induction follows similarly from the fact that $\omega _n \le \lambda _0 = \min D$ Since we are dealing with a club, the limits of the enumeration are limits in $\omega _{n+1}$ of the previous elements, and we can continue the process at limit steps just by taking unions. Finally, for $b: \omega _{n+1} \rightarrow 2$, we let

$$\begin{aligned} \bar{u} ^b = \bigcup _{\lambda \in D} \bar{u} ^{b \restriction \lambda }. \end{aligned}$$

Notice that this implies that for all $\bar{u} \in \textbf{Q}\restriction C^{(n-1)}$ there is a *b* that codes it and for every such *b* we have

$$\begin{aligned} \bar{u} ^{b \restriction \lambda } = \bar{u} ^b \restriction (C \cap P_\lambda )^{(n-1)} \end{aligned}$$

on a club.

Before defining our guessing function, we need the following claim. Such an observation and the consequent definition of *F* seem needed in the argument of [17] as well, yet they are not present.

#### Claim 4.10

Let $\bar{u} \in \textbf{Q}\restriction (C \cap P_\beta )^{(n-1)}$ for some $\beta < \omega _{n+1}$. Then there is at most one $s \in 2^\beta$ such that $\bar{u}$ trivializes $\bar{x} ^s \restriction (C \cap P_\beta )^{(n)}$.

#### Proof of Claim

Assume, towards a contradiction, that there exist distinct *t* and $t'$ such that $\bar{u}$ trivializes both $\bar{x} ^t \restriction (C \cap P_\beta )^{(n)}$ and $\bar{x} ^{t'} \restriction (C \cap P_\beta )^{(n)}$. Let $\alpha \in S^n_{n+1} \cap \beta$ be least such that $t(\alpha ) \ne t'(\alpha )$. Then $\bar{u} \restriction (C \cap P_\alpha )^{(n-1)}$ trivializes $x^t \restriction (C \cap P_\alpha )^{(n)} = x^{t \restriction \alpha } \restriction (C \cap P_\alpha )^{(n)} = x^{t' \restriction \alpha } \restriction (C \cap P_\alpha )^{(n)} = x^{t'} \restriction (C \cap P_\alpha )^{(n)}$.

For the same reasons, $\bar{u} \restriction (C \cap P_{\alpha +1})^{(n-1)}$ trivializes both $x^t \restriction (C \cap P_{\alpha +1})^{(n)} = x^{t \restriction \alpha +1} \restriction (C \cap P_{\alpha +1})^{(n)}$ and $x^{t'} \restriction (C \cap P_{\alpha +1})^{(n)} = x^{t' \restriction \alpha +1} \restriction (C \cap P_{\alpha +1})^{(n)}$. This contradicts property 3 of our construction. $\square$

Then let $T \restriction D$ be the set of all nodes whose level belongs to *D*. We define $F: T \restriction D \rightarrow 2$ as follows. If $\bar{u} ^s$ trivializes $\bar{x} ^{t} \restriction (C \cap P_{\text {dom}(t)})^{(n)}$ for some *t* such that $\text {dom}(s) = \text {dom}(t)$, which is unique by the previous claim, then $F(s) = \epsilon \in \lbrace 0, 1 \rbrace$ such that $\bar{u} ^s$ does not extend to a trivialization of $x^{t, \epsilon }$. Otherwise, let *F*(*s*) take an arbitrary value in $\lbrace 0, 1 \rbrace$.

Now $w \lozenge (S^n_{n+1})$ means precisely that for all $F: 2^{<\omega _{n+1}} \rightarrow 2$ there exists $g: \omega _{n+1} \rightarrow 2$ such that for all $b: \omega _{n+1} \rightarrow 2$.

$$\begin{aligned} S = \lbrace \lambda \in S^n_{n+1} \mid g(\lambda ) = F(b \restriction \lambda ) \rbrace \end{aligned}$$

is stationary. Then $\bar{x} ^g \restriction C^{(n)}$ is nontrivial because for any putative trivialization $\bar{u} ^b$ we get $\lambda \in S \cap D$ such that $g(\lambda ) = F(b \restriction \lambda )$. Now, given the assumption we made towards a contradiction,

$$ \bar{u} ^{b \restriction \lambda } = \bar{u} ^b \restriction (C \cap P_\lambda )^{(n-1)} $$

trivializes

$$\begin{aligned} \bar{x} ^g \restriction (C \cap P_\lambda )^{(n)} = \bar{x} ^{g \restriction \lambda } \restriction (C \cap P_\lambda )^{(n)}. \end{aligned}$$

So, if we use the definition of *F* above for $s= b \restriction \lambda$ and $t = g\restriction \lambda$ we find that cannot be extended to a sequence in $\textbf{Q}\restriction (C \cap P_{\lambda +1} )^{(n-1)}$ that trivializes $\bar{x} ^{g \restriction (\lambda + 1)} \restriction (C \cap P_{\lambda +1} )^{(n)} = \bar{x} ^g \restriction (C \cap P_{\lambda +1} )^{(n)}$, while $\bar{u} ^b \restriction (C \cap P_{\lambda +1} )^{(n-1)}$ is an extension that should do precisely that if $\bar{u} ^b$ is to be a trivialization. $\square$

Finally, we can prove the following.

#### Theorem 4.11

Let $n \ge 1$ and assume that $w \lozenge (S^k_{k+1})$ for $k < n$ and $2^{\aleph _0} < 2^{\aleph _1}$ hold. Then for every directed family $\mathcal {F} \subseteq ({}^\omega \omega , \le ^*)$ containing an $\omega _n$-chain *C* that is unbounded in it and such that $\text {cof} (\mathcal {F}) = \aleph _n$, and for every nontrivial group *G* with $\vert G\vert \le 2^{\aleph _1}$, there exists a coherent family $\bar{x} \in {\textbf{A}^\mathcal {F}_G}^{(n)}$ for *G* such that $\bar{x} \restriction C^{(n)}$ is nontrivial. In particular, $\lim ^n \textbf{A}^\mathcal {F}_G \ne 0$. So, if $\mathfrak {d}= \aleph _n$ and $({}^\omega \omega , \le ^*)$ contains an unbounded chain of length $\omega _n$, then $\lim ^n \textbf{A}_G \ne 0$.

#### Proof

The cardinal arithmetic assumption yields *R*(1, *G*) by Lemma 4.5. Then by the weak diamonds and Lemma 4.9 one can deduce that *R*(*n*, *G*) holds. Then, by Remark 4.8, we deduce that $\lim ^n \textbf{A}^\mathcal {F}_G \ne 0$. for every $\mathcal {F}$ as in the statement of *R*(*n*, *G*). Finally, if $\mathfrak {d} = \aleph _n$ and there exists an unbounded $\omega _n$-chain, then $({}^\omega \omega , \le ^*)$ is among the posets *R*(*n*, *G*) is about. $\square$

#### Remark 4.12

The cardinal arithmetic assumption $2^{\aleph _0} < 2^{\aleph _1}$ is not required for $G = \mathbb {Z}$, i.e. for the pro-group $\textbf{A}$, as it is only needed in the base case for finite groups, as Remark 4.6 shows.

## Cofinal rectangles and weak diamonds

We will now show that all the set-theoretic assumptions that we needed for the proof of the main theorem are relatively consistent with $\textrm{ZFC}$.

First, we need to introduce a “nonlinear" finite support iteration of Hechler’s forcings. The following forcing notion was first introduced in [9].

Let $\mathbb {Q}$ be any well-founded, countably directed poset. Let moreover $\mathbb {Q}^+ = \mathbb {Q} \cup \lbrace *\rbrace$ be the poset that consists of $\mathbb {Q}$ with one more element $*$ on top of it (i.e. greater than all other elements).

For each $a \in \mathbb {Q}^+$, we define a forcing notion $\mathbb {P}_a$. First, let $\mathbb {Q}/a = \lbrace c \in \mathbb {Q} \mid c <_{\mathbb {Q}} a \rbrace$. Suppose now that we have already defined $\mathbb {P}_b$ for all $b <_\mathbb {Q} a$.

1. $p \in \mathbb {P}_a$ if and only if
  - *p* is a finite function with $\textrm{dom}(p) \subseteq \mathbb {Q}/a$.
  - For all $b \in \textrm{dom}(p)$, $p(b) = (t, \dot{g})$ where $t \in {}^{< \omega } \omega$, and $\dot{g}$ is a $\mathbb {P}_b$-name for an element of ${}^\omega \omega$.
2. For $p,q \in \mathbb {P}_a$, we write $p \le q$ if and only if
  - $$ \textrm{dom}(q) \subseteq \textrm{dom}(p) $$.
  - For all $b \in \textrm{dom}(q)$, if $p(b) = (s,\dot{f})$ and $q(b) = (t,\dot{g})$ then
    (i) $$t = s \restriction \textrm{dom}(t) $$
    (ii) $$ p \restriction (\mathbb {Q} / b) \Vdash _{\mathbb {P}_b} \textrm{dom}(t) \le n < \textrm{dom}(s) \Rightarrow s(n) > \dot{g} (n) $$.
    (ii) $$ p \restriction (\mathbb {Q} / b) \Vdash _{\mathbb {P}_b} \forall n \,\, \dot{f} (n) \ge \dot{g} (n) $$.

The following is the case $\lambda = \omega$ of [6, Theorem 1].

### Theorem 5.1

Suppose $\mathbb {Q}$ is any countably directed well-founded poset. Let $\mathbb {H}_{\mathbb {Q}} = \mathbb {P}_*$ be the poset corresponding to the maximum $*$ of $\mathbb {Q}^+$ as in the definition above.

1. $$\mathbb {H}_\mathbb {Q} $$ is ccc.
2. $V^{\mathbb {H}_\mathbb {Q}} \models \mathbb {Q}$ embeds cofinally into $( {}^\omega \omega , <^*)$.
3. If $V \models$ “ the bounding number of $\mathbb {Q}$ is $\mu$" then $V^{\mathbb {H}_\mathbb {Q}} \models \mathfrak {b}= \mu$.
4. If $V \models$ “ the dominating number of $\mathbb {Q}$ is $\nu$" then $V^{\mathbb {H}_\mathbb {Q}} \models \mathfrak {d}= \nu$.

We now focus on the specific instance of Hechler’s forcing that we are going to need for our consistency result.

### Proposition 5.2

$\mathbb {H}_{\omega _k \times \omega _n}$ is $\kappa$-Knaster for every uncountable regular cardinal $\kappa$.

### Proof

Let *A* be a set of conditions of size $\kappa$. By the $\Delta$-system lemma, we can thin out to an $A' \subseteq A$ with $\vert A' \vert = \kappa$ such that the supports of the conditions in $A'$ form a $\Delta$-system. Then we thin out to $A'' \subseteq A'$, again with $\vert A'' \vert = \kappa$, and such that the stems of all conditions in the root of the $\Delta$-system are equal. Now for any two $q_0, q_1 \in A''$ we find $p \le q_0, q_1$ as follows. Let $\text {dom}(p) = \text {dom}(q_0) \cup \text {dom}(q_1)$ and $p(b) = (s_b, \dot{f}_b)$ when $q_i(b) = (s_b, \dot{g}^i_b)$ for $i \in \lbrace 0, 1 \rbrace$. Finally, by the existential completeness lemma we can choose names such that $\mathbbm {1}_{\mathbb {P}_b} \Vdash \dot{f}_b \ge \dot{g}^i_b$ for $i \in \lbrace 0,1 \rbrace$. $\square$

### Proposition 5.3

Let $k \le n < \omega$. If $V \models \textrm{CH}$ then $\mathbb {H}_{\omega _k \times \omega _n}$ has size $\aleph _n$ and $V^{\mathbb {H}_{\omega _k \times \omega _n}} \models \mathfrak {c}= \aleph _n$.

### Proof

Let $(\omega _k, \omega _n) = *$ be the maximum element of the associated poset. One proves by mutual transfinite induction on the well-founded order relation that for all $(\omega _1, \omega _1) \le (\alpha , \beta ) \le (\omega _k \times \omega _n)$ we have $\vert \mathbb {P}_{(\alpha , \beta )} \vert = \max \lbrace \vert \alpha \vert , \vert \beta \vert \rbrace$ and $V^{\mathbb {P}_{(\alpha , \beta )}} \models \mathfrak {c}= \max \lbrace \vert \alpha \vert , \vert \beta \vert \rbrace$. One implication follows from the fact that the nonlinear iteration is finite support and the other follows by the technique of nice names [10, Lemma IV 3.11]. $\square$

This allows us to apply the following lemma.

### Lemma 5.4

(see [17]) Let $\kappa$ be an uncountable regular cardinal, and let $\lambda$ and $\mu$ be cardinals such that $\lambda ^{< \kappa } < \mu$. Suppose $\mathbb {P}$ is a $\kappa$-cc poset of size $\lambda$. Let $\mathbb {Q} = \textrm{Fn}(\mu \times \kappa , 2, \kappa )$, that is, the poset of partial functions from $\mu \times \kappa$ to 2 of size strictly less than $\kappa$. Then, for every stationary subset *S* of $\kappa$ in *V*, $w \lozenge (S)$ holds in the generic extension by $\mathbb {P} \times \mathbb {Q}$.

In fact, Lemma 5.4 allows us to prove that a certain forcing extension satisfies all of our requirements.

### Theorem 5.5

Let $k, n \le \omega$. Then it is relatively consistent with $\textrm{ZFC}$ that there is a cofinal set in ${}^\omega \omega$ isomorphic to $\omega _k \times \omega _n$ with respect to $<^*$, that $2^{\aleph _0} < 2^{\aleph _1}$, and $w \lozenge (S^i_{i+1})$ for all $1 \le i < n$.

### Proof

We start with a ground model $V \models \text {GCH}$. For $1 \le i \le n$, let $\mathbb {C}_i = \textrm{Fn}(\omega _{n+i} \times \omega _i, 2, \omega _i)$ and $\mathbb {C} = \mathbb {H}_{\omega _k \times \omega _n} \times \mathbb {C}_1 \times ... \times \mathbb {C}_n$ be the product forcing notion. Let $G = G_0 \times ... \times G_n$ be a generic filter over $\mathbb {C}$. It is well known [10, Lemma V.2.6] that $\mathbb {C}_1 \times ... \times \mathbb {C}_n$ preserves cofinalities; moreover, it is $\sigma$-closed and so does not add any reals. Hence, by the product lemma [10, Theorem V.1.2] $V[G] = V[G_1 \times ... \times G_n][G_0]$ and so we have indeed a cofinal subset of shape $\omega _k \times \omega _n$ in the extension, as well as preservation of cofinalities and $2^{\aleph _i} = \aleph _{n+i}$ for $0 \le i \le n$ (again, by nice names) as $\mathbb {H}_{\omega _k \times \omega _n}$ is absolute between *V* and $V[G_1 \times ... \times G_n]$.

As for $w \lozenge (S^i_{i+1})$, note that for all $i < n$ the notion $\mathbb {C}_{i+2} \times ... \times \mathbb {C}_n$ is $\omega _{i+2}$-closed. Hence if we let $W = V[G_{i+2} \times ... \times G_n]$ we have $2^{\aleph _i} = \aleph _{i+1}$ in *W*. Now we apply Lemma 5.4 over *W* with $\mathbb {P} = \mathbb {H}_{\omega _k \times \omega _n} \times \mathbb {C}_1 \times ... \times \mathbb {C}_i$, $\mathbb {Q} = \mathbb {C}_{i+1}$, $\kappa = \omega _{i+1}$, $\lambda = \omega _{n+i}$, $\mu =\omega _{n+i+1}$ , and $S = S^i_{i+1}$. Here $\mathbb {P}$ is $\kappa -cc$ because the factors are $\kappa$-Knaster; this is well-known for the generalized Cohen forcings and has been proved for $\mathbb {H}_{\omega _k \times \omega _n}$ in Proposition 5.2. We get the conclusion again by the product lemma. $\square$

### Proof of Main Theorem

Theorem 5.5 together with 4.11 easily implies the consistency of the statement $\lim ^n \textbf{A}_G \ne 0$ with the relevant values of $\mathfrak {b}$ and $\mathfrak {d}$. The case $G = \mathbb {Z}$ is precisely the Main Theorem. $\square$

## Open Questions

### Question 6.1

Can the condition $\vert G \vert \le 2^{\aleph _1}$ be removed from Lemma 4.9 and subsequent results?

### Question 6.2

Is $\lim ^n \textbf{A}\ne 0$ for $n>1$ consistent with the failure of $\bigwedge _{1 \le i <n } w \lozenge (S^i_{i+1})$?

## Data Availability

No datasets were generated or analysed during the current study.
